# Development and validation of an interpretable stacking-based risk model for breast cancer- related lymphoedema: a cross-sectional study

**DOI:** 10.3389/fonc.2026.1844242

**Published:** 2026-05-29

**Authors:** Ke-Ming Ying, Cheng-Xue Dang, Qing Zhou, Han Xue

**Affiliations:** 1 Department of Surgical Oncology, The Central Hospital of Hanzhong, Hanzhong, Shaanxi, China; 2 Department of Surgical Oncology, The First Affiliated Hospital of Xi’an Jiaotong University, Xi’an, Shaanxi, China

**Keywords:** breast cancer-related lymphedema, interpretable model, postoperative behaviors characteristics, risk prediction model, stacking ensemble learning

## Abstract

**Background:**

Breast Cancer-Related Lymphedema (BCRL) is one of the common complications after breast cancer treatment. It is characterized by irreversibility and high disability. Currently, early identification of high-risk patients remains a clinical pain point. This study focuses on BCRL risk prediction, integrating machine learning techniques with multi-dimensional clinical data to construct a practical prevention and treatment tool, providing a reference for precise prevention and treatment of BCRL.

**Methods:**

Patients who underwent breast cancer surgery and treatment from January 2021 to May 2025 were included. The diagnostic criterion for BCRL was “the difference in upper limb circumference ≥2 cm”. Using LASSO regression, Support Vector Machine Recursive Feature Elimination (SVM-REF), Random Forest (RF) and Boruta, 34 variables were subjected to feature selection, and features selected by all four algorithms were used as modeling variables. The SHAP value analysis was performed to construct and validate an interpretable BCRL risk prediction model using a Stacking ensemble learning approach, and a visual Web-based risk prediction tool was developed.

**Results:**

A total of 570 eligible patients were included. They were stratified into the training set (420 cases) and the temporal validation set (150 cases). Seven core characteristics were selected, including clinical characteristics (BMI, tumor clinical stage, surgical method, axillary lymph node handling method and diabetes) and modifiable characteristics (patients’ awareness of BCRL and the time spent holding their mobile phones daily after surgery). The Stacking model performed the best, featuring high accuracy, high precision and interpretability. The ROC-AUC values for the training and validation sets were 0.911 and 0.868, respectively, and the PR-AUC values were 0.857 and 0.789, respectively. The Web tool, StackBCRL, can be accessed online.

**Conclusions:**

Through the construction of an interpretable BCRL risk prediction model based on Stacking ensemble learning by integrating postoperative behavioral characteristics (the time spent holding their mobile phones daily after surgery and patients’ awareness of BCRL), the developed BCRL risk assessment system achieved real-time individualized risk prediction and visualization. It provides a simple and practical method for large-scale, low-cost early screening of BCRL, is suitable for promoting clinical application and provides an innovative solution for BCRL prevention and control.

## 1 Introduction

With the widespread adoption of early screening methods and the continuous advancement of multidisciplinary treatment strategies such as surgery, radiotherapy, chemotherapy, targeted therapy and immunotherapy, the early diagnosis rate of breast cancer has significantly improved, and treatment outcomes have also improved, resulting in a substantial increase in patient survival rates. At present, the 5-year survival rate for breast cancer patients in developed countries exceeds 85% (1, 2), and in some countries, it is greater than 90% (1, 3). Even in developing countries with relatively limited medical resources, the 5-year survival rate can reach approximately 60% (1). The high survival rate has transformed breast cancer from a fatal disease into a chronic disease; however, difficulties and challenges for survivors in terms of long-term management remain. Studies have shown that more than 60% of breast cancer survivors suffer from at least one treatment-related complication (4). Breast cancer-related lymphedema (BCRL) severely affects the quality of life of breast cancer patients because of its irreversibility and high disability rate (5–7).

BCRL is among the most common and serious complications following breast cancer treatment. It occurs primarily because of surgical procedures such as axillary lymph node dissection (ALND) and sentinel lymph node biopsy (SLNB), which cause damage to the lymphatic vessels, resulting in impaired lymphatic fluid return and subsequently leading to excessive accumulation of interstitial fluid in the upper limbs, causing edema (8). BCRL not only manifests as swelling of the affected upper limb but also may be accompanied by symptoms such as pain, functional impairment, and skin changes, which severely affect the daily life and mental health of patients (7). As the global burden of breast cancer and the survival period of patients with this disease increase, BCRL has become an important public health issue and is attracting increasing attention (9).

Many studies have confirmed that precision medicine and risk stratification management methods play assist in preventing BCRL (5). By accurately identifying high-risk patients with BCRL and formulating individualized prevention strategies, early detection and intervention can be achieved, thereby effectively reducing the incidence of BCRL and improving the quality of life of patients. Moreover, such strategies can be used to optimize the allocation of medical resources, with the limited prevention and monitoring resources predominantly used for high-risk groups, thereby increasing service efficiency and cost effectiveness. BCRL risk prediction models can accurately identify patients who require comprehensive intervention and play crucial roles in improving clinical decisions. Furthermore, this type of model is highly important for the education of breast cancer patients, as it can help patients and their families better understand the risks of BCRL, improve their understanding of preventive measures, and increase their compliance with preventive measures.

At present, the existing BCRL risk prediction models still have problems such as high bias and insufficient generalizability, making it difficult for such models to fully meet the actual needs of precise prediction and early intervention in clinical practice (10). Most studies focus on uncontrollable high-risk factors for BCRL (such as age, body mass index (BMI), comorbidities, tumor clinical stage, surgical method, radiotherapy and chemotherapy). In contrast, controllable high-risk factors, including postoperative behaviors and lifestyle habits, have received little attention.

With the rapid development of mobile phone entertainment, breast cancer patients often hold their mobile phones for entertainment purposes for prolonged periods after surgery. Previous studies have indicated that the prolonged use of mobile phones for entertainment may lead to health problems (11–14). However, in current clinical practice, the specific association between this lifestyle habit and the occurrence of BCRL has not been clearly defined, and it is unclear whether changing such postoperative behaviors and lifestyles has a positive effect on the symptoms of BCRL. Moreover, traditional risk prediction models have inherent limitations, resulting in insufficient accuracy in identifying high-risk populations for BCRL and an inability to provide reliable support for early clinical interventions. On this basis, in this study, the stacking ensemble learning method was applied to construct a BCRL risk prediction model, and the performance of the model and its clinical application value were systematically evaluated.

The aim of this study is to construct a reliable and interpretable BCRL risk prediction model on the basis of traditional uncontrollable risk factors. By clarifying the association of BCRL with postoperative behaviors and lifestyle habits, especially the daily time spent holding mobile phones, this model will provide a practical tool and a decision-making basis for early clinical screening and individualized prevention of BCRL. This will also help healthcare professionals identify patients at high risk of BCRL early, guide such patients to optimize their postoperative behaviors and lifestyle habits, and apply targeted preventive measures. Ultimately, this model will reduce the risk of BCRL, alleviate patient suffering and economic burden, and improve the quality of life of patients after breast cancer surgery.

## 2 Materials and methods

A cross-sectional design was adopted in this study, with the occurrence of BCRL in patients as the binary outcome variable (yes/no). The patients were recruited among outpatients and inpatients in the departments of Oncology Surgery, Oncology Internal Medicine, Rehabilitation Medicine, Pain Management, and Anesthesiology of Hanzhong Central Hospital from January 2021 to May 2025.

The inclusion criteria were as follows: (1) female patients aged 18 years or older; (2) those diagnosed with unilateral breast cancer after January 2015; (3) patients who had undergone axillary surgery (including ALND or axillary SLNB); (4) those who had undergone breast cancer treatment (surgery, radiotherapy or chemotherapy) for at least 3 months; (5) those whose clinical stage of breast cancer was I to III and whose estimated survival period was ≥1 year; and (6) those who were conscious and able to complete the questionnaire survey and physical examination.

Patients who met any of the following criteria were excluded: (1) were pregnant or breastfeeding; (2) had not previously undergone breast cancer surgery; (3) had other malignant tumors or abnormal functions of important organs; (4) had not completed the prescribed radiotherapy and/or chemotherapy regimens; (5) had not completed the prescribed radiotherapy regimen and had other underlying diseases that may have caused edema (such as congestive heart failure, kidney diseases, and malnutrition); (6) had a significant trauma, surgical history or infection history in the upper limbs or neck; (7) had incomplete clinical data for the upper limbs, with insufficient information for effective data analysis; (8) were currently participating in other clinical trials that might have affected the results of this study; and (9) were unable to complete the research questionnaire.

According to the above criteria, a total of 570 patients were included. The 420 patients enrolled from January 2021 to December 2023 were used as the training set for model construction and internal validation; the 150 patients enrolled from January 2024 to May 2025 were used as the temporal validation set to evaluate the generalization ability of the model. Because the validation set originated from the same institution, this is not an external validation but a temporal validation. The temporal validation set was used to objectively evaluate the model’s performance on future data from the same center.

### 2.1 Study variables

In this study, a data collection form consisting of five sections and a total of 34 variables was employed (**Table 1**). The first four sections included demographic characteristics, clinical and pathological features of breast cancer, treatment methods, comorbidities and postoperative complications, which were extracted from the electronic medical records system. The fifth section addresses postoperative behavioral and cognitive factors related to BCRL risk. The core variable, “daily mobile phone use time after surgery”, is collected through the following question: “During the waking state, approximately how much time do you spend using the affected hand or both hands to actively hold the mobile phone for browsing, typing, gaming, etc. (excluding placing the phone on the table or passive carrying)? “. Response options were: (1) <1 hour; (2) 1–2 hours; (3) >2 hours. The question instructed patients to report time when the affected upper limb was actively engaging in holding the phone. The cut-offs were determined based on preliminary analysis of the training set, which showed a non-linear increase in BCRL risk: compared with <1 hour/day, the unadjusted odds ratio for 1-2 hours was 1.8 (95% CI: 1.1–2.9), and for >2 hours it was 3.9 (95% CI: 2.3–6.6). Although no formal reliability or validity assessment was conducted, the questionnaire was pilot-tested on 30 breast cancer patients to check for clarity and comprehension; minor wording adjustments were made based on patient feedback.” The data of patients who underwent surgery in our hospital were obtained from the electronic medical records system, whereas for patients from other hospitals, the information was collected through structured questionnaires via WeChat, WeChat mini-programs, phone calls, or outpatient follow-ups. After fully explaining the purpose and significance of the research to the participants or their legal guardians, the subjects independently completed the questionnaire after signing the informed consent form.

**Table 1:** Classification of variables used to predict the risk of BCRL collected in this study.

Category	Variables
Demographic characteristics	Ethnicity, age, BMI, marital status, educational level, average monthly family income
Clinical and pathological features of breast cancer	Position of the affected side, whether it is the dominant hand, location of the tumor, clinical stage of the tumor, T stage of the tumor, N stage of the tumor, pathological type, and molecular subtype
Treatment methods	Surgical method, treatment method for axillary lymph nodes, level of axillary lymph node dissection, neoadjuvant chemotherapy, postoperative chemotherapy, postoperative radiotherapy, radiotherapy area, endocrine therapy, targeted therapy
Comorbidity and postoperative complications	Diabetes, hypertension, nonhypertensive cardiovascular diseases, thyroid disorders, postoperative complications
Postoperative behavioral lifestyle	Patient's awareness of BCRL, postoperative rehabilitation care, postoperative smoking, postoperative drinking, staying up late postoperatively, and postoperative time spent holding a mobile phone

To increase the reliability and validity of the self-reported behavioral variables, we designed a self-compiled structured questionnaire to collect data on behaviors and lifestyle habits, including the patient’s awareness of BCRL, postoperative rehabilitation care, postoperative smoking, postoperative drinking, staying up late after surgery, and postoperative time spent using mobile phones. The questionnaire items were developed on the basis of a systematic review of previously published studies and relevant clinical guidelines, aiming to reduce the risk of BCRL (15, 16).

The questionnaire survey was performed jointly by two researchers who were trained and qualified: one was responsible for asking the questions, while the other was responsible for recording the answers and supervising. The researchers checked the data in real time to ensure its accuracy and completeness. After the questionnaire was completed, the participants reviewed the information onsite and could immediately indicate any problems, which were corrected by the researchers. This ensured the authenticity and reliability of the data through mutual confirmation between the participants and the researchers.

### 2.2 Assessment of BCRL

**Figure 1:**
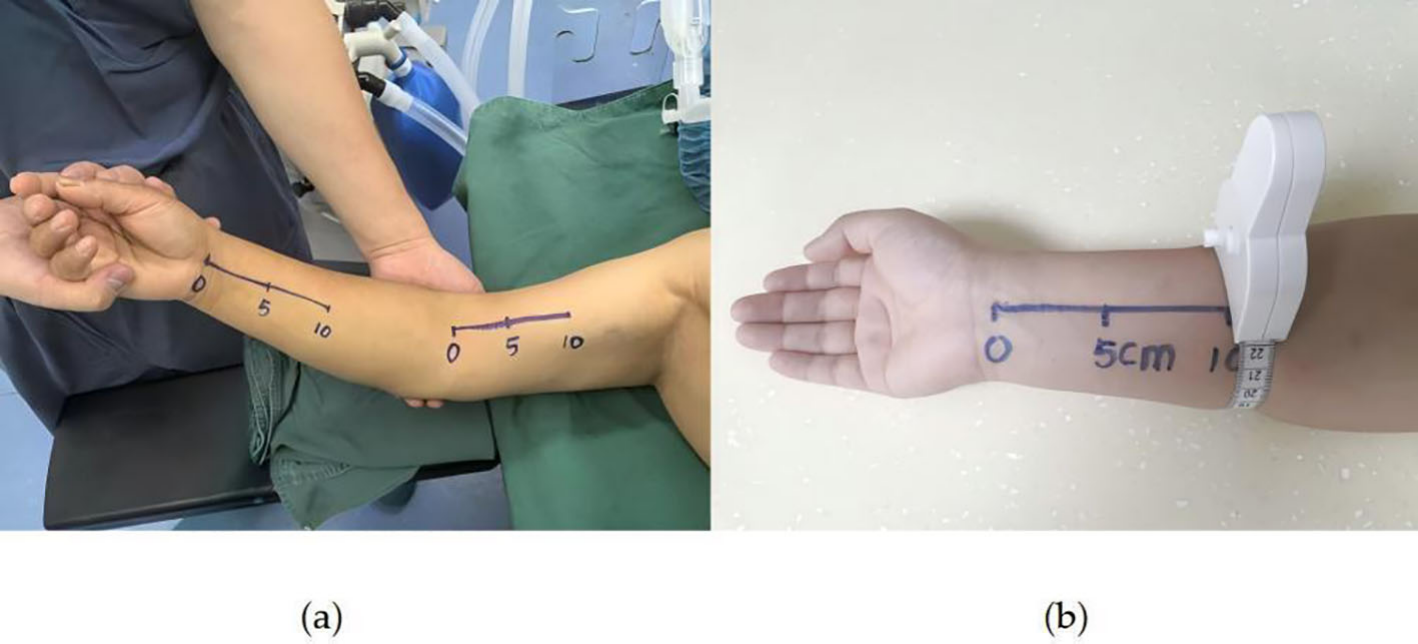
Images of the upper limb circumference measurement process: **(a)** measurement points; **(b)** measurement of the corresponding arm circumference.

The patient’s awareness of BCRL, postoperative rehabilitation care, postoperative smoking, and postoperative alcohol consumption were collected. In this study, an upper arm circumference difference of ≥2 cm was used as the core diagnostic criterion for BCRL (17). The specific assessment process was as follows. Using an automatic precise measuring circumference ruler, the four parts of the patient’s bilateral upper limbs (the wrist crease, 10cm above the wrist crease, the anterior elbow fossa, and 10cm above the anterior elbow fossa) were measured simultaneously using the 4-point measurement method (**Figure 1**). If the difference in arm circumference between the affected and healthy sides of the body for any part was ≥2 cm and if there were related clinical symptoms (such as limb swelling, pain, or limited mobility), BCRL was comprehensively diagnosed. All affected areas were assessed by professionally trained medical staff who underwent unified training in accordance with standardized operating procedures at the time of patient enrollment, thereby ensuring the accuracy and consistency of the assessment results.

### 2.3 Data preprocessing and feature selection

Continuous variables such as age and BMI were discretized to eliminate the differences in units among the variables and to reduce the sensitivity of some ML algorithms to the scale of the variables. For variables such as age and BMI, as well as unordered categorical variables, one-hot encoding was used to convert each category into an independent binary variable to avoid introducing artificial order bias. For ordered categorical variables, numerical encoding was performed on the basis of the inherent order of the categories.

Among the 34 candidate variables, missing data were minimal (range 0%–4.2%, all variables<5%). Because missingness appeared random, we performed complete-case analysis (n=570). A sensitivity analysis using k-nearest neighbors imputation (k=5) gave nearly identical model performance (AUC difference<0.01), supporting the robustness of the approach.

In terms of feature selection, a consensus strategy involving multiple methods was adopted to increase the robustness and significance of the selected features. On the basis of 10-fold stratified cross-validation, LASSO regression, support vector machine-recursive feature elimination (SVM-REF), random forest (RF), and Boruta algorithms were applied for feature selection. All feature selection procedures were performed exclusively on the training set (n=420). The temporal validation set (n=150) was kept completely untouched until the final model evaluation. No information from the validation set was used at any stage of feature selection, hyperparameter tuning, or model selection. Finally, the features selected by all four methods were used as the modeling variables.

### 2.4 Construction of the stacking ensemble learning model

A stacking strategy was adopted to construct the integrated model. Stacking is a hierarchical stacking ensemble learning method. The lower layer consists of different types of base learners, and each base learner is trained independently and outputs preliminary prediction results on the basis of the input data; the upper layer is the meta-model, which receives the outputs of all the lower models as a new feature set and then provides the final prediction.

Eight typical machine learning algorithms, namely, logistic regression (LR), naive Bayes, K-nearest neighbors (KNN), RBF-SVM, multilayer perceptron (MLP), decision tree (DT), random forest (RF), and XGBoost, were selected as the base models. Using the area under the receiver operating characteristic (ROC) curve (AUC) as the performance metric, the hyperparameters were optimized through a combination of random grid search and Bayesian optimization. The number of iterations was set to 50, and the initial number of sampling points was 2. To address the class imbalance issue in the BCRL dataset, the SMOTE oversampling technique (18) was introduced to preprocess the data, thereby alleviating the interference of class bias in the training process. SMOTE oversampling was applied strictly within the 10-fold stratified cross-validation on the training set. Specifically, for each fold, SMOTE was used only on the training sub-fold to generate synthetic minority class samples; the validation sub-fold was never oversampled. This prevents data leakage and yields an unbiased performance estimate. After hyperparameter tuning, the final model was retrained on the entire training set after SMOTE. The predicted probability values of the base model based on the training set were used as the input features, and the ROC-AUC was used as the performance evaluation metric. Finally, a meta-model (i.e., the final stacking ensemble model) was constructed via LASSO regression.

### 2.5 Model validation and indicator evaluation

With the ROC-AUC as the core evaluation metric, a 10-fold stratified cross-validation method was employed to assess the stability and consistency of the model’s performance on the training data. To simulate the application scenarios of the model in real clinical environments, a temporal validation strategy was adopted to construct the validation set. This strategy is based on the core principle of time independence. After the training set period was selected, new patient data that were consistent with the source and scenario of the training set were collected to form the validation set, thereby simulating the clinical application paradigm of “modeling based on historical data to predict future cases”. Specifically, the data of 150 patients from January 2024 to May 2025 were selected as the temporal validation set which was no overlap at all with the training set (January 2021 to December 2023) to objectively evaluate the generalizability of the model for new data in the future.

The performance of the model was comprehensively evaluated by using multiple indicators. The ROC-AUC and the area under the precision–recall curve (PR-AUC) were used to evaluate discriminability. Moreover, the accuracy rate, sensitivity and specificity are reported. In terms of calibration, the Brier score and calibration curves were used to evaluate the consistency between the predicted probabilities and the actual risk occurrence. In terms of clinical practicability, the clinical net benefit under different decision thresholds was quantified using the DCA model.

### 2.6 Model explanation

In this study, the Shapley value explanation (SHAP) method (19) was employed to conduct an attributable analysis of the BCRL risk prediction model. This method provides explanations at both the global and local levels. The global explanations are provided by ranking the importance of the features, revealing the overall correlation between each feature and the risk of BCRL; the local explanations show the contribution of specific individual features to the risk of BCRL, clarifying their individualized risk status.

### 2.7 Development of a visual web tool

In this study, the R/Shiny framework was used to develop web-based visualization applications. An interactive web application was developed based on the R/Shiny framework to visualize the BCRL risk prediction model and interpretability analysis results. This application consists of the following four core modules: (1) a data input interface, which offers a structured form that enables users to enter the relevant risk data of patients online; (2) a prediction engine, which uses the pretrained stacking ensemble model to generate real-time BCRL risk prediction results; (3) a result output module, which provides the predicted probability and BCRL risk classification for individual patients in a structured format; and (4) a visual explanation module, which integrates the SHAP analysis method and presents the contribution of each feature to the prediction results through interactive force diagrams and waterfall charts, thereby revealing the decision-making mechanism of the BCRL risk model.

### 2.8 Statistical analysis

Quantitative data that follow a normal distribution are expressed as *x ± s*, and comparisons between groups were conducted using the independent sample *t* test; nonnormally distributed quantitative data are presented as the median and interquartile range, and comparisons between groups were performed using the Mann–Whitney U test; and categorical data are expressed as the number (%) of cases, and comparisons between groups were conducted using the χ^2^ test. The Delong test was used to compare the differences in the ROC-AUC values among the different models and to evaluate the discriminability of the models. *P*<0.05 was considered to indicate statistical significance. The data analysis was conducted in R (version 4.5.0). The main R packages used include tidyverse, tidymodels, stacks, ggplot2, fastshap, shapviz and shiny.

## 3 Results

### 3.1 Participant characteristics

This study ultimately included 570 breast cancer patients. The data were divided into a training set (420 patients) and an temporal validation set (150 patients) according to the time stratification principle. There was no significant difference in the incidence of BCRL between the two groups: 37.1% in the training set and 36.7% in the validation set (*P*>0.05). The demographic characteristics, clinical and pathological features of the tumors, treatment methods, comorbidities, postoperative complications, postoperative behaviors and lifestyle habits and other characteristics were evenly distributed between the training and temporal validation sets. The baseline characteristics of the two groups of patients were highly comparable. The results reveal that multiple characteristics are significantly associated with the occurrence of BCRL.

In terms of demographic characteristics, the BMI of the patients in the BCRL group in the training set was significantly greater than that of patients in the non-BCRL group, and the BMI of the patients in the BCRL group in the temporal validation set was also significantly greater than that of the patients in the non-BCRL group (*P*<0.01) (**Table 2**).

**Table 2:** Comparative analysis of the demographic characteristics of the patients in the BCRL group and the non-BCRL group.

Variable	Training set (n=420)			Validation set (n=150)			Overall *P* value
	BCRL group (n=156)	Non-BCRL group (n=264)	*P* value	BCRL group (n=55)	Non-BCRLgroup (n=95)	*P* value	
Etdnicity			0.793			0.669	0.185
Han etdnicity	151 (96.8%)	258 (97.7%)		51 (92.7%)	91 (95.8%)		
Otder etdnic	5 (3.2%)	6 (2.3%)		4 (7.3%)	4 (4.2%)		
Age (years)			0.481			0.016	0.237
<40	25 (16.0%)	35 (13.3%)		15 (27.3%)	10 (10.5%)		
40~60	113 (72.4%)	189 (71.6%)		34 (61.8%)	78 (82.1%)		
>60	18 (11.5%)	40 (15.2%)		6 (10.9%)	7 (7.4%)		
BMI (kg/m²)			<0.001			<0.001	0.427
Underweight	4 (2.6%)	27 (10.2%)		1 (1.8%)	6 (6.3%)		
Normal	58 (37.2%)	130 (49.2%)		16 (29.1%)	61 (64.2%)		
Overweight	71 (45.5%)	79 (29.9%)		29 (52.7%)	22 (23.2%)		
Obesity	23 (14.7%)	28 (10.6%)		9 (16.4%)	6 (6.3%)		
Marital status			0.428			0.581	0.657
Unmarried	5 (3.2%)	10 (3.8%)		3 (5.5%)	3 (3.2%)		
Married	137 (87.8%)	219 (83.0%)		48 (87.3%)	84 (88.4%)		
Divorce	5 (3.2%)	18 (6.8%)		3 (5.5%)	3 (3.2%)		
Widowed	9 (5.8%)	17 (6.4%)		1 (1.8%)	5 (5.3%)		
Educational level			0.420			0.319	0.183
Primary school and below	15 (9.6%)	17 (6.4%)		2 (3.6%)	2 (2.1%)		
Middle School	73 (46.8%)	127 (48.1%)		32 (58.2%)	44 (46.3%)		
High/Vocational School	51 (32.7%)	80 (30.3%)		17 (30.9%)	34 (35.8%)		
College degree or above	17 (10.9%)	40 (15.2%)		4 (7.3%)	15 (15.8%)		
Per capita montdly income (RMB)			0.067			0.126	0.591
<1000	59 (37.8%)	124 (47.0%)		25 (45.5%)	39 (41.1%)		
1000~2000	68 (43.6%)	102 (38.6%)		18 (32.7%)	46 (48.4%)		
2000~4000	22 (14.1%)	35 (13.3%)		8 (14.5%)	8 (8.4%)		
>4000	7 (4.5%)	3 (1.1%)		4 (7.3%)	2 (2.1%)		

In terms of the clinical pathological characteristics of breast cancer, the differences in tumor clinical stage, tumor T stage, tumor N stage, and tumor pathological type between the training and validation sets were statistically significant (*P*<0.01). The differences in tumor clinical stage, tumor T stage, tumor N stage, and tumor pathological type between the BCRL and the non-BCRL groups in the temporal validation set were also statistically significant (*P*<0.05) (**Table 3**).

**Table 3:** Comparative analysis of the clinical and pathological characteristics of the tumors of the patients in the BCRL group and the non-BCRL group.

Variable	Training set (n=420)			Validation set (n=150)			Overall *P* value
	BCRL group (n=156)	Non-BCRL group (n=264)	*P* value	BCRL group (n=55)	Non-BCRL group (n=95)	*P* value	
Affected position			0.501			0.541	0.162
Left	79 (50.6%)	144 (54.5%)		23 (41.8%)	46 (48.4%)		
Right	77 (49.4%)	120 (45.5%)		32 (58.2%)	49 (51.6%)		
Main arm			0.851			0.692	0.837
No	84 (53.8%)	146 (55.3%)		31 (56.4%)	49 (51.6%)		
Yes	72 (46.2%)	118 (44.7%)		24 (43.6%)	46 (48.4%)		
Tumor location			0.142			0.265	0.418
Outer upper quadrant	51 (32.7%)	98 (37.1%)		19 (34.5%)	35 (36.8%)		
Outer lower quadrant	25 (16.0%)	55 (20.8%)		9 (16.4%)	10 (10.5%)		
Inner upper quadrant	23 (14.7%)	43 (16.3%)		5 (9.1%)	17 (17.9%)		
Inner lower quadrant	27 (17.3%)	23 (8.7%)		5 (9.1%)	14 (14.7%)		
Central district	12 (7.7%)	19 (7.2%)		9 (16.4%)	7 (7.4%)		
Nipple area	18 (11.5%)	26 (9.8%)		8 (14.5%)	12 (12.6%)		
Clinical staging			<0.001			<0.001	0.881
I	11 (7.1%)	117 (44.3%)		2 (3.6%)	45 (47.4%)		
II	104 (66.7%)	122 (46.2%)		39 (70.9%)	43 (45.3%)		
III	41 (26.3%)	25 (9.5%)		14 (25.5%)	7 (7.4%)		
T stage			<0.001			<0.001	0.948
T1	46 (29.5%)	163 (61.7%)		15 (27.3%)	63 (66.3%)		
T2	37 (23.7%)	44 (16.7%)		12 (21.8%)	14 (14.7%)		
T3	47 (30.1%)	40 (15.2%)		19 (34.5%)	13 (13.7%)		
T4	26 (16.7%)	17 (6.4%)		9 (16.4%)	5 (5.3%)		
N stage			<0.001			0.019	0.580
N0	51 (32.7%)	136 (51.5%)		21 (38.2%)	54 (56.8%)		
N1	76 (48.7%)	111 (42.0%)		24 (43.6%)	37 (38.9%)		
N2	9 (5.8%)	6 (2.3%)		2 (3.6%)	1 (1.1%)		
N3	20 (12.8%)	11 (4.2%)		8 (14.5%)	3 (3.2%)		
Patdological type			<0.001			0.002	0.580
Invasive ductal carcinoma	115 (73.7%)	153 (58.0%)		43 (78.2%)	48 (50.5%)		
Invasive lobular carcinoma	15 (9.6%)	61 (23.1%)		9 (16.4%)	24 (25.3%)		
Otder	26 (16.7%)	50 (18.9%)		3 (5.5%)	23 (24.2%)		
Molecular typing			0.636			0.598	0.886
HER2 positive	35 (22.4%)	56 (21.2%)		11 (20.0%)	20 (21.1%)		
Luminal A	54 (34.6%)	104 (39.4%)		18 (32.7%)	35 (36.8%)		
Luminal B1	5 (3.2%)	6 (2.3%)		4 (7.3%)	2 (2.1%)		
Luminal B2	55 (35.3%)	92 (34.8%)		21 (38.2%)	35 (36.8%)		
Triple-negative	7 (4.5%)	6 (2.3%)		1 (1.8%)	3 (3.2%)		

In terms of the treatment methods, the surgical approach, axillary lymph node handling method, axillary lymph node dissection level, NAC, and radiotherapy area were significantly correlated with the occurrence of BCRL in the training set (*P*<0.01), and the surgical approach, axillary lymph node handling method, axillary lymph node dissection level, NAC, and radiotherapy area were significantly correlated with the occurrence of BCRL in the temporal validation set (*P*<0.01) (**Table 4**).

**Table 4:** Comparison of treatment methods between the patients in the BCRL group and non-BCRL group.

Variable	Training set (n=420)			Validation set (n=150)			Overall *P* value
	BCRL group (n=156)	Non-BCRL group (n=264)	*P* value	BCRL group (n=55)	Non-BCRL group (n=95)	*P* value	
Surgical metdod			<0.001			<0.001	0.382
Breast-conserving	24 (15.4%)	149 (56.4%)		4 (7.3%)	51 (53.7%)		
Total excision	132 (84.6%)	115 (43.6%)		51 (92.7%)	44 (46.3%)		
Treatment for ALN			<0.001			<0.001	0.072
ALND	128 (82.1%)	125 (47.3%)		41 (74.5%)	36 (37.9%)		
SLNB	28 (17.9%)	139 (52.7%)		14 (25.5%)	59 (62.1%)		
ALN dissection level			<0.001			<0.001	0.078
I	28 (17.9%)	139 (52.7%)		14 (25.5%)	59 (62.1%)		
I~II	114 (73.1%)	113 (42.8%)		31 (56.4%)	34 (35.8%)		
I~III	14 (9.0%)	12 (4.5%)		10 (18.2%)	2 (2.1%)		
NAC			<0.001			<0.001	1.000
No	72 (46.2%)	201 (76.1%)		23 (41.8%)	75 (78.9%)		
Yes	84 (53.8%)	63 (23.9%)		32 (58.2%)	20 (21.1%)		
Adjuvant chemotderapy			0.172			0.231	0.715
No	86 (55.1%)	126 (47.7%)		33 (60.0%)	46 (48.4%)		
Yes	70 (44.9%)	138 (52.3%)		22 (40.0%)	49 (51.6%)		
Radiotderapy			0.660			0.280	0.954
No	6 (3.8%)	14 (5.3%)		1 (1.8%)	7 (7.4%)		
Yes	150 (96.2%)	250 (94.7%)		54 (98.2%)	88 (92.6%)		
Radiotderapy area			<0.001			<0.001	0.153
No	6 (3.8%)	14 (5.3%)		1 (1.8%)	7 (7.4%)		
Full breast	24 (15.4%)	149 (56.4%)		4 (7.3%)	51 (53.7%)		
Tdoracic wall	74 (47.4%)	65 (24.6%)		36 (65.5%)	28 (29.5%)		
Tdoracic wall+Supraclavicular/inferior cervical region	52 (33.3%)	36 (13.6%)		14 (25.5%)	9 (9.5%)		
Endocrine tderapy			0.502			0.894	0.811
No	42 (26.9%)	62 (23.5%)		12 (21.8%)	23 (24.2%)		
Yes	114 (73.1%)	202 (76.5%)		43 (78.2%)	72 (75.8%)		
Targeted tderapy			0.823			1.000	0.852
No	66 (42.3%)	116 (43.9%)		23 (41.8%)	40 (42.1%)		
Yes	90 (57.7%)	148 (56.1%)		32 (58.2%)	55 (57.9%)		

In terms of comorbidities and potoperative complications, there were significant differences in diabetes status, hypertension status, and postoperative complications between the BCRL and the non-BCRL groups in the training set (*P*<0.05). Among them, the proportions of patients with diabetes in the BCRL and non-BCRL groups were 25.6% and 13.3%, respectively. The occurrence of diabetes, hypertension, and postoperative complications in the temporal validation set was significantly correlated with the occurrence of BCRL (*P*<0.05). Among them, the proportions of patients with diabetes in the BCRL and non-BCRL groups were 32.7% and 15.8%, respectively (**Table 5**).

**Table 5:** Comparison of comorbidities and postoperative complications between the patients in the BCRL group and non-BCRL group.

Variable	Training set (n=420)			Validation set (n=150)			Overall *P* value
	BCRL group (n=156)	Non-BCRL group (n=264)	*P* value	BCRL group (n=55)	Non-BCRL group (n=95)	*P* value	
Diabetes			0.002			0.027	0.322
No	116 (74.4%)	229 (86.7%)		37 (67.3%)	80 (84.2%)		
Yes	40 (25.6%)	35 (13.3%)		18 (32.7%)	15 (15.8%)		
Hypertension			0.027			0.036	0.111
No	103 (66.0%)	144 (54.5%)		43 (78.2%)	57 (60.0%)		
Yes	53 (34.0%)	120 (45.5%)		12 (21.8%)	38 (40.0%)		
Nonhypertensive cardiovascular diseases			0.298			0.543	0.156
No	105 (67.3%)	163 (61.7%)		41 (74.5%)	65 (68.4%)		
Yes	51 (32.7%)	101 (38.3%)		14 (25.5%)	30 (31.6%)		
Tdyroid disorders			0.195			0.212	0.165
No	134 (85.9%)	239 (90.5%)		43 (78.2%)	83 (87.4%)		
Yes	22 (14.1%)	25 (9.5%)		12 (21.8%)	12 (12.6%)		
Postoperative complications			0.046			0.002	0.515
No	116 (74.4%)	219 (83.0%)		38 (69.1%)	86 (90.5%)		
Yes	40 (25.6%)	45 (17.0%)		17 (30.9%)	9 (9.5%)		

In terms of postoperative behaviors and lifestyle habits, there were significant differences between the two groups in terms of the patient’s awareness of BCRL, postoperative rehabilitation care, and the duration spent holding a mobile phone each day after surgery (*P*<0.05). The proportion of patients who held a mobile phone for more than 2 hours per day in the BCRL group was 68.6%, which was significantly greater than that in the other time groups. The cognitive level, postoperative rehabilitation care, and time spent holding a mobile phone each day after surgery were significantly correlated with the occurrence of BCRL in the temporal validation group (*P*<0.05). The proportion of patients who held a mobile phone for more than 2 hours per day in the BCRL group was 70.9%, which was significantly greater than that in the other time groups (**Table 6**).

**Table 6:** Comparison of the postoperative behaviors and lifestyle habits of patients in the BCRL group and the non-BCRL group.

Variable	Training set (n=420)			Validation set (n=150)			Overall *P* value
	BCRL group (n=156)	Non-BCRL group (n=264)	*P* value	BCRL group (n=55)	Non-BCRL group (n=95)	*P* value	
Patient's awareness of BCRL			0.008			0.013	0.200
General	21 (13.5%)	13 (4.9%)		12 (21.8%)	5 (5.3%)		
Not knowing	45 (28.8%)	66 (25.0%)		17 (30.9%)	32 (33.7%)		
Medium	45 (28.8%)	88 (33.3%)		14 (25.5%)	24 (25.3%)		
Very good	45 (28.8%)	97 (36.7%)		12 (21.8%)	34 (35.8%)		
Postoperative rehabilitation care			0.023			0.004	0.476
General	17 (10.9%)	16 (6.1%)		10 (18.2%)	3 (3.2%)		
Medium	22 (14.1%)	22 (8.3%)		10 (18.2%)	11 (11.6%)		
Bad	60 (38.5%)	135 (51.1%)		24 (43.6%)	48 (50.5%)		
Very good	57 (36.5%)	91 (34.5%)		11 (20.0%)	33 (34.7%)		
Postoperative smoking			0.515			0.508	0.585
Never	151 (96.8%)	249 (94.3%)		52 (94.5%)	93 (97.9%)		
Occasionally	3 (1.9%)	9 (3.4%)		1 (1.8%)	1 (1.1%)		
Frequently	2 (1.3%)	6 (2.3%)		2 (3.6%)	1 (1.1%)		
Postoperative drinking			0.475			0.318	0.147
Never	124 (79.5%)	196 (74.2%)		37 (67.3%)	67 (70.5%)		
Occasionally	31 (19.9%)	66 (25.0%)		18 (32.7%)	25 (26.3%)		
Frequently	1 (0.6%)	2 (0.8%)		0 (0%)	3 (3.2%)		
Postoperative staying up late			0.574			0.510	0.615
Never	77 (49.4%)	142 (53.8%)		28 (50.9%)	46 (48.4%)		
Occasionally	46 (29.5%)	76 (28.8%)		20 (36.4%)	30 (31.6%)		
Frequently	33 (21.2%)	46 (17.4%)		7 (12.7%)	19 (20.0%)		
Postoperative time spent holding a mobile phone (h/d)			0.002			0.040	0.438
<1	6 (3.8%)	36 (13.6%)		0 (0%)	10 (10.5%)		
1~2	43 (27.6%)	83 (31.4%)		16 (29.1%)	28 (29.5%)		
>2	107 (68.6%)	145 (54.9%)		39 (70.9%)	57 (60%)		

### 3.2 Feature selection

In this study, multiple ML feature selection methods were integrated to construct a robust and interpretable BCRL risk prediction model. Specifically, four algorithms, namely, Boruta, LASSO regression, RF, and SVM-RFE, were employed for multiple validations and variable selection. Ultimately, these algorithms were used to determine the final predictive variables to be included in the model. The highly important features identified by the four feature selection algorithms vary (**Table 7**).

**Table 7:** The feature selection results of the four methods.

Algorithm	Selecting features	Number
Boruta	Surgical method, clinical stage of the tumor, radiotherapy area, T stage of the tumor, daily time spent holding a mobile phone after surgery, handling method of axillary lymph nodes, level of axillary lymph node dissection, BMI, NAC, coexisting hypertension, coexisting diabetes, tumor pathological type, patient's awareness of BCRL, N stage of the tumor, coexisting nonhypertensive cardiovascular diseases	15
LASSO	BMI, educational level, tumor quadrant location, tumor clinical stage, surgical method, axillary lymph node handling method, postoperative complications, coexisting diabetes, coexisting hypertension, coexisting nonhypertensive cardiovascular disease, patient's awareness of BCRL, postoperative rehabilitation care, postoperative smoking, postoperative alcohol consumption, postoperative daily mobile phone usage time	15
RF	Radiotherapy area, surgical method, clinical stage of the tumor, handling method of axillary lymph nodes, level of axillary lymph node dissection, tumor T stage, daily time spent holding a mobile phone after surgery, BMI, NAC, tumor pathological type, coexistence of hypertension, patient's awareness of BCRL, presence of nonhypertensive cardiovascular diseases, tumor N stage, coexistence of diabetes, tumor quadrant location, postoperative rehabilitation care	17
SVM-RFE	Radiotherapy area, clinical stage of the tumor, surgical method, tumor T stage, level of axillary lymph node dissection, method of handling axillary lymph nodes, NAC, tumor N stage, BMI, patient's awareness of BCRL, presence of diabetes, daily time spent holding a mobile phone after surgery, tumor pathological type, postoperative complications, tumor quadrant location	15
Feature intersection	Surgical method, clinical stage of the tumor, daily time spent holding a mobile phone after surgery, method of handling axillary lymph nodes, BMI, coexistence of diabetes, patient's level of awareness of BCRL	7

**Figure 2:**
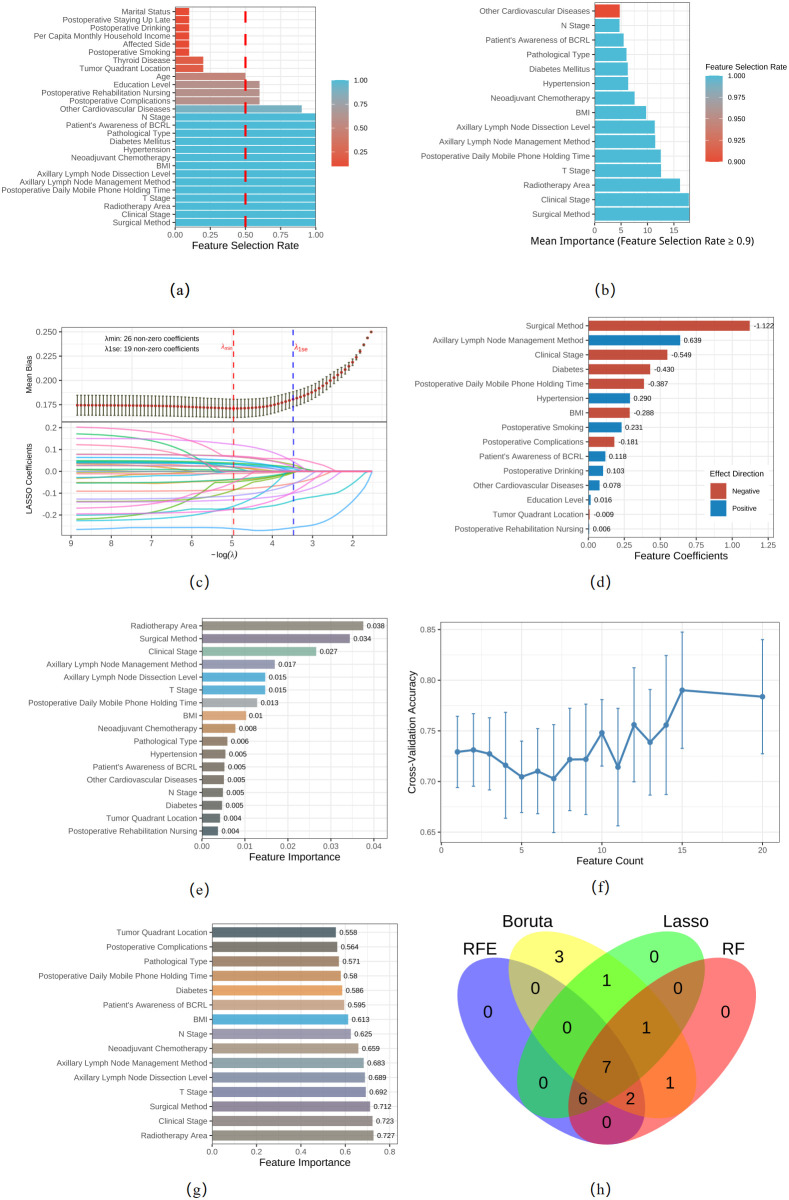
The results of the four feature selection algorithms: **(a)** feature selection frequency of the Boruta algorithm; **(b)** importance of the 9 features selected by the Boruta algorithm; **(c)** LASSO regression feature selection cross-validation curve (top) and coefficient path (bottom); **(d)** LASSO regression of the feature coefficients; **(e)** feature selection results using the RF algorithm; **(f)** tenfold cross-validation feature results; **(g)** the importance of the features determined by the SVM-RFE algorithm; **(h)** the intersection of the features selected by the four algorithms.

The results of the four feature selection algorithms reveal significant differences in the assessment of feature importance: The Boruta, LASSO regression and SVM-RFE models all selected 15 features, whereas the RF model selected 17 features. The Boruta model identified a total of 15 features, with features such as the surgical method, clinical stage of the tumor, radiotherapy area, T stage of the tumor, and daily time spent using a mobile phone having relatively high selection rates (**Figure 2a**) and importance (**Figure 2b**), whereas features such as combined hypertension, combined diabetes, and pathological type had relatively lower selection rates. The LASSO regression model also selected 15 features, with the surgical method, axillary lymph node handling method, and tumor clinical stage identified as the most important (**Figures 2c, d**). The RF algorithm identified a total of 17 features, which was the most by any model. The most important features included the radiotherapy area, surgical method, tumor clinical stage, and axillary lymph node handling method (**Figure 2e**). The SVM-RFE and 10-fold cross-validation approach revealed that when the number of features was 15, the model’s cross-validation accuracy was the highest (**Figure 2f**). Among them, features such as the radiation therapy area, tumor clinical stage, and surgical method were particularly important (**Figure 2g**).

A multimethod consensus strategy was employed in this study, integrating the recognition results of the above four algorithms and selecting the commonly identified features. On the basis of the consensus strategy, 7 core modeling features were ultimately identified (**Table 2**; **Figure 2h**), covering various clinical factors, such as demographic characteristics (BMI), tumor clinical pathological features (clinical stage), treatment methods (surgical method, axillary lymph node handling method), comorbidities (diabetes), and postoperative behaviors and lifestyle factors (patient’s awareness of BCRL, daily mobile phone usage time after surgery). These features comprehensively covered the main factors influencing the occurrence of BCRL.

### 3.3 Construction and evaluation of the optimal model

**Figure 3:**
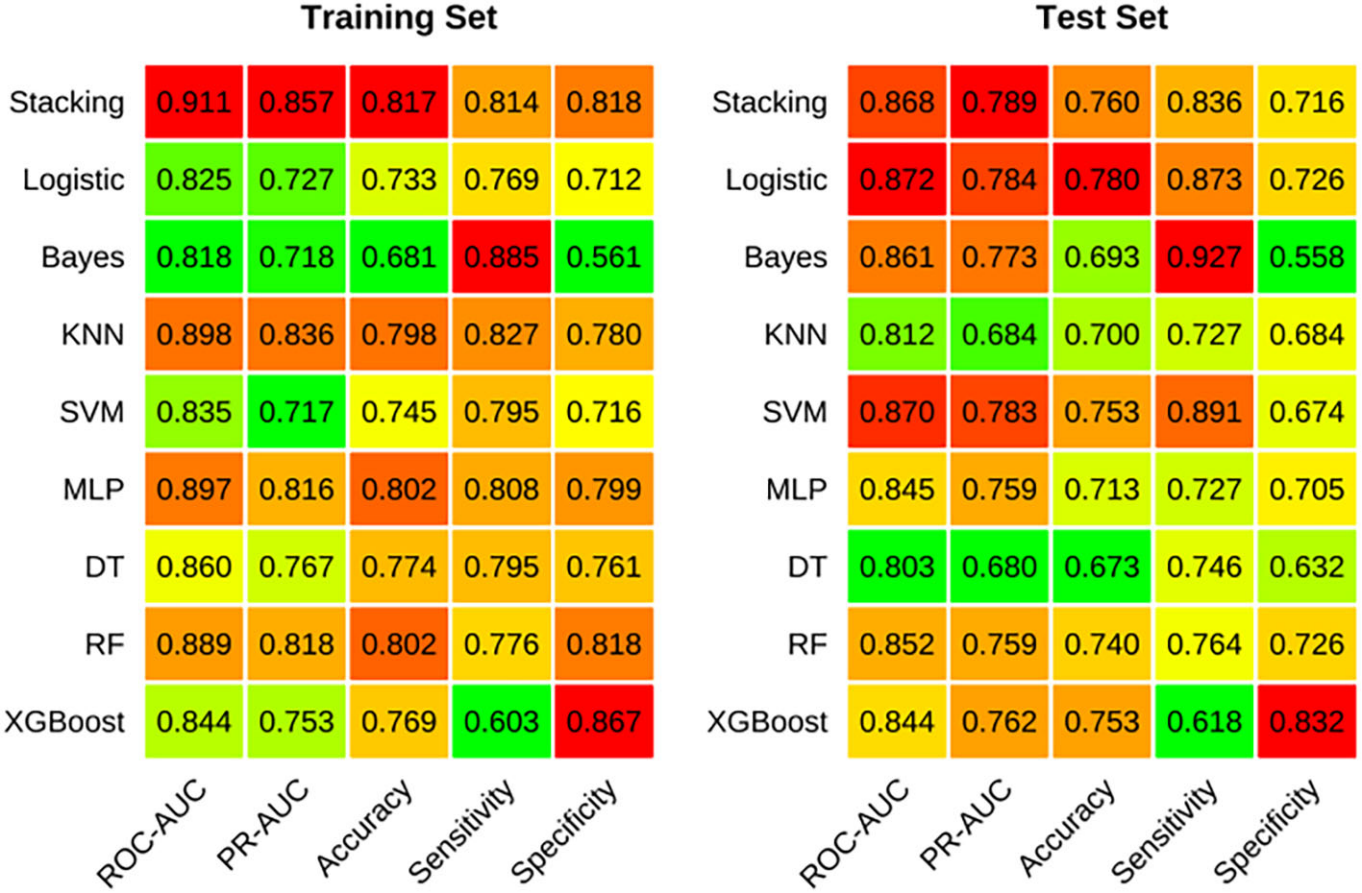
Comparison of the performance indicators of the 9 prediction models on the training set (left) and the validation set (right).

On the training set, the Stacking ensemble model achieved an ROC-AUC of 0.911, a PR-AUC of 0.857, an accuracy of 0.817, a sensitivity of 0.814, and a specificity of 0.818 (left side of **Figure 3**). Its overall performance was superior to that of the single models, such as the LR model, naive Bayes model, KNN model, SVM model, MLP model, DT model, RF model, and XGBoost model. The majority of its performance metrics were better than those of the single models. Some individual models show obvious imbalances in performance metrics: (1) the Naive Bayes model had a sensitivity of 0.885, but its specificity was only 0.561, indicating a weak ability to identify negative samples; (2) the XGBoost model had a specificity of 0.867, but its sensitivity was only 0.603, suggesting an insufficient ability to identify positive samples; (3) the KNN model, MLP model, and RF model perform similarly to the stacking ensemble model in terms of some individual metrics, but their overall performance, considering all the metrics, still lags behind that of the stacking ensemble model.

On the validation set, although the performance of the stacking ensemble model slightly declined compared with that on the training set, its overall performance still demonstrated good stability and was competitive (right side of **Figure 3**): the ROC-AUC was 0.868 (95% CI: 0.823–0.906); the PR-AUC was 0.789 (95% CI: 0.731–0.842), remaining at a relatively high level; the sensitivity reached 0.836 (95% CI: 0.765–0.895), indicating outstanding performance; and the specificity was 0.716 (95% CI: 0.642–0.788); and the Brier score was 0.154 (95% CI: 0.128–0.182). The LR model achieved an ROC-AUC of 0.780 (95% CI: 0.732–0.824), PR-AUC of 0.720 (95% CI: 0.668–0.771), accuracy of 0.760 (95% CI: 0.688–0.822), sensitivity of 0.745 (95% CI: 0.662–0.818), specificity of 0.758 (95% CI: 0.688–0.822), and Brier score of 0.154 (95% CI: 0.130–0.180). Compared with each individual model, the stacking ensemble model demonstrates a more balanced performance. The LR model (0.780) was slightly superior to the stacking ensemble model (0.760) in terms of accuracy, and its PR-AUC was also close to that of the stacking model. Moreover, there was no significant difference in sensitivity or specificity between the two models. Although the overall performance of the two models was comparable, the LR model does not have a clear advantage. The sensitivity (0.927) of the naive Bayes model was extremely high, but the specificity (0.558) was insufficient. Although the SVM model had a high sensitivity (0.891), its specificity (0.674) was relatively low, indicating a significant imbalance in performance.

**Figure 4:**
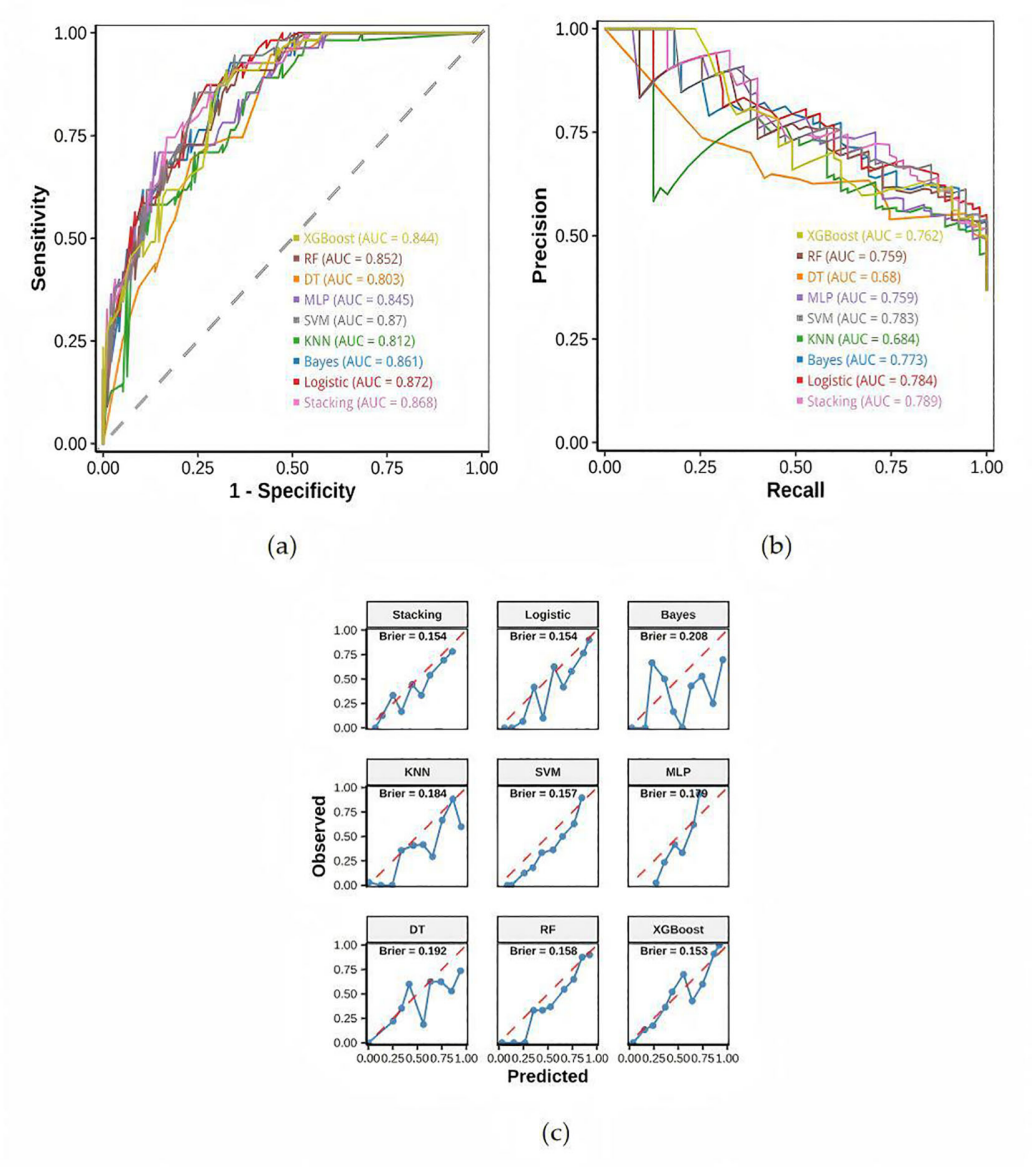
The ROC curves, PR curves and calibration curves of the 9 models on the validation set: **(a)** ROC curve; **(b)** PR curve; **(c)** calibration curve.

**Figure 5:**
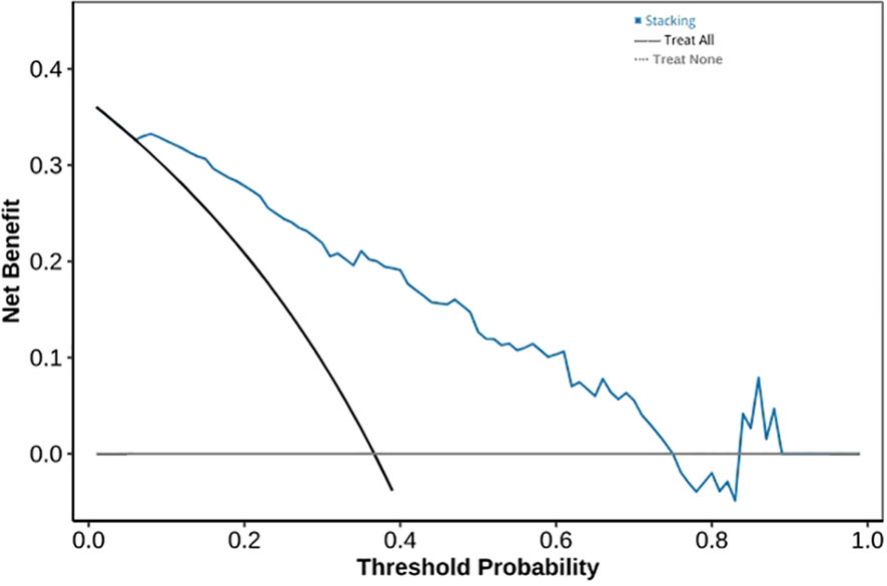
DCA results of the stacking ensemble learning model.

The ROC curve, PR-AUC curve and calibration plot of the model on the validation set are presented in **Figure 4**. In terms of the calibration performance, the stacking integrated model and the LR model have the lowest Brier scores (both 0.154), and their predicted probabilities are the closest to the actual results. However, the naive Bayes model (Brier=0.208) has a poorer calibration performance than the other models. As shown in **Figure 5**, within the wide threshold range of 0 to 0.8, the DCA of the stacking ensemble model consistently shows a stable positive net improvement.

### 3.4 Model explanation

**Figure 6:**
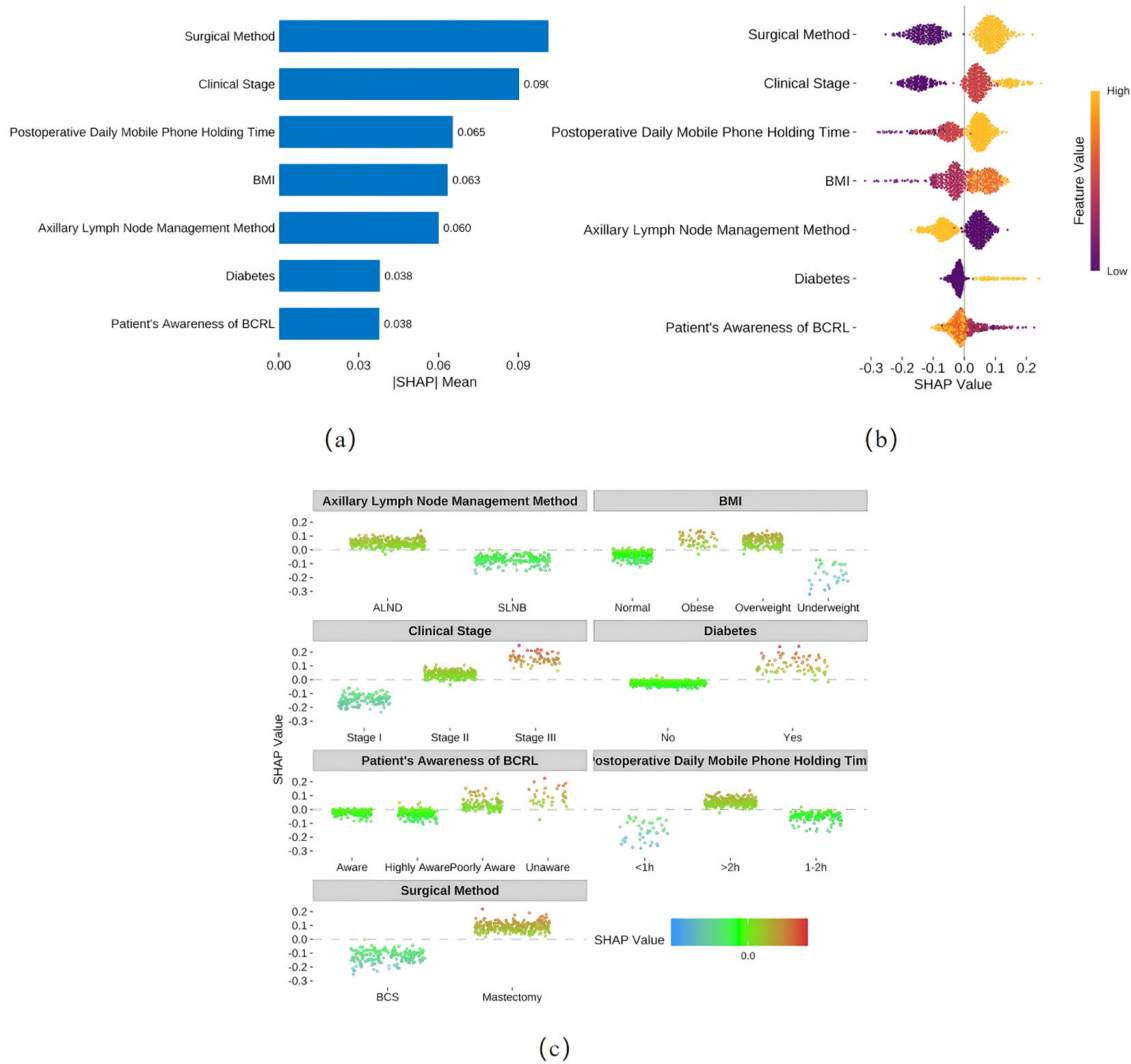
Stacking-based SHAP feature analysis of the ensemble learning models: **(a)** SHAP feature importance bar chart; **(b)** SHAP feature importance swarm plot; **(c)** SHAP feature importance dependency graph.

The results of the stacking ensemble model were explained according to the SHAP values, and a bar chart (**Figure 6a**) and a swarm plot (**Figure 6b**) of the importance of the SHAP features were constructed. The SHAP feature importance bar chart shows that the importance of each feature in predicting the risk of BCRL is clearly stratified. The surgical methods (0.105) and clinical tumor staging (0.090) were significantly more important than the other features. The daily mobile phone usage time after surgery (0.065), BMI (0.063), and axillary lymph node treatment method (0.060) were secondary risk factors, and diabetes status (0.038) and the patient’s level of awareness of BCRL (0.038) were relatively less important.

The SHAP feature importance swarm plot reveals the influence of each feature on the prediction results of the BCRL risk model. When the feature values are at the “risk end” (for example, the surgical method is total mastectomy, the clinical stage of the tumor is stage III, and the daily time spent holding a mobile phone after surgery is more than 2 hours), the SHAP values are mostly positive, indicating that the risk of BCRL is greater for this feature combination.

The SHAP feature dependence plot (**Figure 6c**) further explains the individual impact of each feature on the risk of BCRL occurrence. The SHAP values for total mastectomy surgery are mostly positive, while those for breast-conserving surgery are mostly negative. The SHAP feature dependency graph (**Figure 6c**) further reveals that for each feature, the SHAP value transitions from positive to negative as the clinical stage of the BCRL tumor is identified earlier (from stage III to stage II to stage I). The SHAP value is mostly positive when the time spent holding a mobile phone daily is more than 2 hours after surgery, mostly negative when the time is less than 1 hour, and in an intermediate range when the time is 1 to 2 hours. The SHAP values of overweight and obese breast cancer patients are mostly positive, while those of normal and underweight breast cancer patients are mostly negative. The SHAP values of ALND patients are mostly positive, while those of SLNB patients are mostly negative. The SHAP values of breast cancer patients with diabetes are mostly positive, while those of breast cancer patients without diabetes are mostly negative. In terms of the patient’s level of understanding of BCRL, the SHAP values of those who were “not familiar” or “not very familiar” were mostly positive, while the SHAP values of those who were “familiar” or “very familiar” were mostly negative.

**Figure 7:**
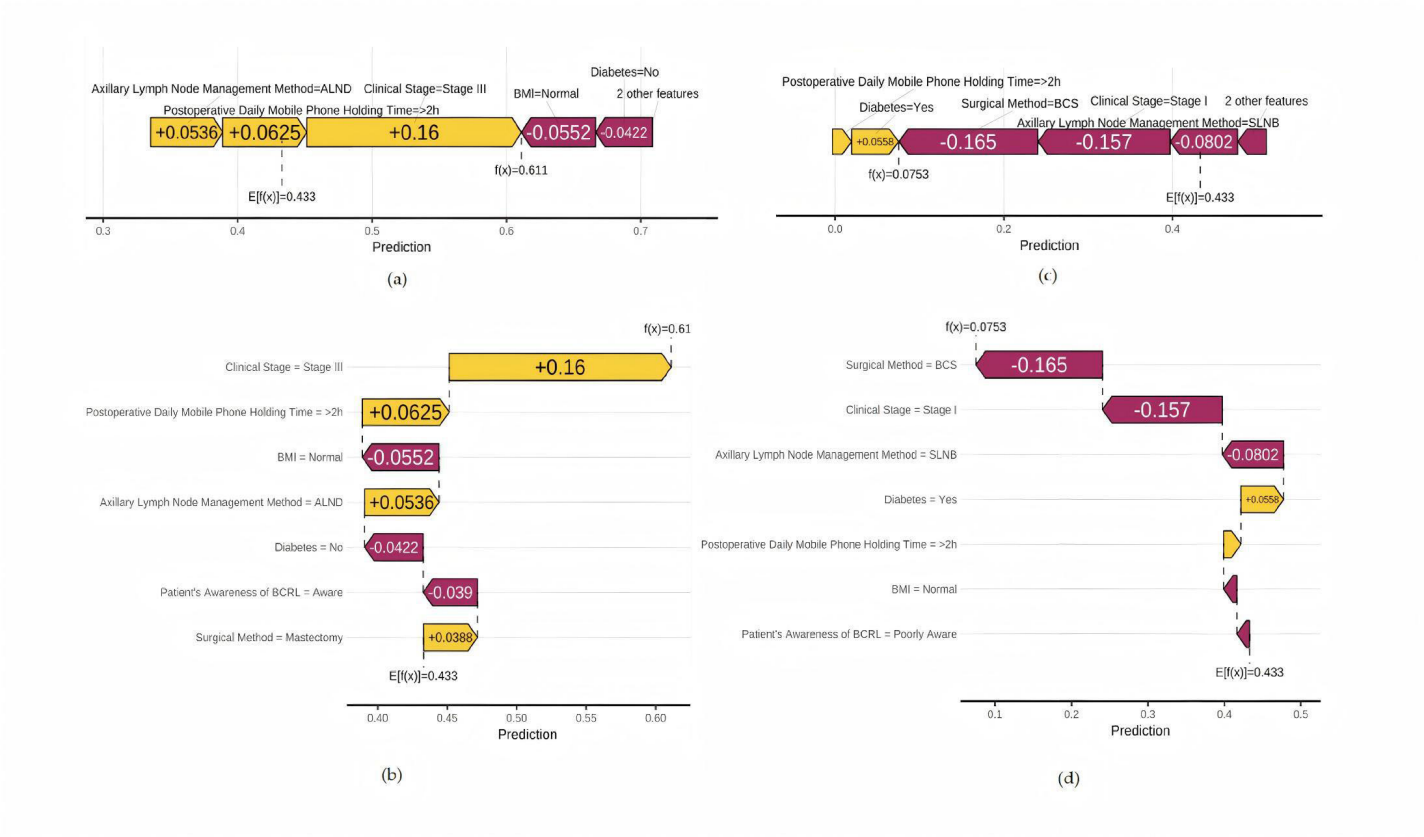
SHAP force plot and feature contribution waterfall chart for positive samples (BCRL patients) and negative samples (non-BCRL patients): **(a)** single positive SHAP force plot; **(b)** single positive sample waterfall chart; **(c)** single negative SHAP force plot; **(d)** single negative sample waterfall chart.

The SHAP force diagrams and waterfall charts of a BCRL patient and a non-BCRL patient are shown in **Figure 7**. In the SHAP force plot of the positive samples (**Figure 7a**), ALND, mobile phone use for more than 2 hours per day after surgery, and a stage III classification had positive SHAP values, whereas the SHAP values for normal weight and no diabetes were negative. In the waterfall chart of the positive samples (**Figure 7b**), the model performs predictions starting from the baseline value (E[f(x)] = 0.433), successively adds the SHAP values of each feature, and finally obtains a prediction probability of 0.611 for this patient. The results clearly show the cumulative contribution of each feature in the prediction process.

The SHAP analysis results of the negative samples reveal different characteristic influence patterns compared with those of the positive samples. In the SHAP force plot (**Figure 7c**) and the waterfall plot (**Figure 7d**), breast-conserving surgery, a stage I classification, and SLNB have negative SHAP values, whereas daily mobile phone usage for more than 2 hours after surgery and diabetes have positive SHAP values.

### 3.5 Visual web tool: StackBCRL

On the basis of the R/Shiny framework, the BCRL risk assessment system StackBCRL was developed. This system includes three main modules: (1) The data management module supports single data entry and batch import/export. It covers clinical indicators related to BCRL, such as height, weight, surgical method, tumor clinical stage, axillary lymph node handling method, history of diabetes, level of awareness of BCRL, and daily mobile phone usage time. It provides standardized data templates to ensure accurate input. (2) The risk assessment module integrates a pretrained stacking ensemble model, automatically converts clinical indicators into model features, outputs individual BCRL risk probabilities and binary classification results, supports batch prediction and provides real-time feedback on processing progress and statistical summaries. (3) The interpretability analysis module generates feature contribution maps and waterfall charts on the basis of the SHAP algorithm to visually display the influence direction and intensity of each clinical indicator on the individual risk prediction of BCRL. Additionally, this module exports the analysis results for research reports. StackBCRL uses shiny dashboard to construct a structured interface, provides Chinese interactive guidance, and is designed to meet the needs of clinical researchers. StackBCRL is deployed via the Shiny Web platform (https://dbf5yo-aikemi-xia.shinyapps.io/StackBCRL/), facilitating communication among peers and promoting research on BCRL risk assessment and prevention.

## 4 Discussion

In this study, BCRL risk prediction was systematically explored. The two main results are as follows. First, a BCRL risk prediction model based on a stacking ensemble learning method was constructed and validated. This model integrates postoperative behavioral and lifestyle characteristics, including the daily time spent holding a mobile phone after surgery and the patient’s level of awareness of BCRL. Second, a BCRL risk assessment web application (StackBCRL) was developed on the basis of the R/Shiny framework. The stacking ensemble learning model has excellent performance, and its evaluation indicators during the training stage are outstanding. In the testing stage, it shows good generalizability and performance, and its overall performance is significantly better than that of all the individual base models. The data confirm that this risk prediction model can accurately identify patients with a high risk of BCRL. StackBCRL enables real-time prediction and visualization of individualized risks; provides a simple, efficient and practical tool for large-scale, low-cost early screening of BCRL; and has good clinical application value.

The stacking ensemble learning model constructed in this study is based on clinical and pathological features that have been clinically validated, providing a solid theoretical foundation for the application of the model in clinical settings. The core risk factors identified by the model are highly consistent with the conclusions of previous studies, further verifying the reliability of the model. The results of the model clearly indicate that surgical scope is a decisive risk factor for the occurrence of BCRL. ALND and total mastectomy are associated with the highest risk and were identified as the most important features. These results are supported by the findings of multiple large-scale clinical studies and systematic reviews (8, 20–23). Moreover, the model results confirmed that a more advanced clinical stage of breast cancer is a key risk factor for BCRL, which is consistent with the results of Shen et al. (8), Renet et al. (20), and Wuraola et al. (24). Furthermore, the model revealed a high body mass index (indicating metabolic-related risk) as an independent predictor of BCRL, and this conclusion has been verified in the literature (25–28).

The key finding of this study is that various postoperative behavioral and lifestyle factors are associated with the occurrence of BCRL. In particular, longer daily mobile phone use time after surgery was identified as a factor associated with higher BCRL risk. However, because of the cross-sectional design, this association should not be interpreted as causal. The observed association may be hypothesized to involve certain behavioral patterns, such as prolonged low-intensity static muscle activity of the fingers and wrist (an ‘inefficient muscle pump’ state) or sustained poor posture compressing axillary and subclavian lymphatic pathways (29–31). It is also possible that patients who spend more time on their phones might be more likely to neglect early warning signals from the affected limb (32, 33). However, these mechanisms remain speculative. Furthermore, we cannot exclude residual confounding by unmeasured factors, including general physical inactivity, precise arm posture, rehabilitation compliance, and socioeconomic status. Therefore, this finding should be viewed as hypothesis-generating rather than conclusive, and prospective studies are needed to test causality.

In recent years, modifiable behavioral factors and related cognitive aspects of BCRL have attracted increasing clinical attention (34–36). The incorporation of such controllable factors into the BCRL risk prediction model has become an important direction in current clinical research. This also reflects the development trend of BCRL risk research, which is shifting from uncontrollable factors to controllable factors, with the research depth gradually increasing. The results of this study revealed high-risk factors for BCRL among postoperative behavioral factors. In particular, the risk associated with longer durations of mobile phone use during the postoperative period was identified for the first time. This breakthrough not only serves as an important supplement to early models focused on traditional clinical and pathological factors but also represents an advancement in BCRL risk research. This study, through a data-driven approach, clearly revealed the independent predictive value of postoperative behavioral and lifestyle factors on the risk of BCRL. These results can effectively expand the application scope of existing BCRL risk prediction models, further improve BCRL risk assessment systems, and provide practical references for research on controllable factors.

The proposed stacking ensemble learning model and the StackBCRL visualization tool were developed based on a solid theoretical foundation and clear clinical logic. Their value involves both the good model performance and their clinical applicability, and the three core aspects are as follows. First, the main advantage of the stacking integrated model is its potential use in clinical applications. This model integrates complementary base learners to reduce the bias and variance of the individual models, thereby increasing its generalizability and robustness. The results on the validation set show that the model has excellent comprehensive performance (ROC-AUC=0.868, PR-AUC=0.789, sensitivity=0.836, and specificity=0.716), and all the indicators are superior to those of the individual base learners. This represents a successful clinical application of the principle of ensemble learning. Second, the model has high interpretability, and the results of the model are highly consistent with clinical pathological mechanisms, which is critical to its clinical applicability. The SHAP analysis revealed that the risk factors identified by the model are consistent with medical knowledge, the importance of the features is clearly stratified, and individual predictions can be visualized to enable transparent and traceable decision-making. The results confirm that the proposed model is not merely a data fitting tool but also a clinical abstraction of the pathogenesis of BCRL, significantly increasing its credibility and acceptance in clinical settings. Finally, the StackBCRL visualization tool enables the practical application of the research results in clinical settings. By integrating core functions and simplifying operations, this tool has a lower usage threshold and can provide direct support for clinical assessments and patient interventions. Its online deployment also facilitates sharing and iterative updates, marking a crucial advance from academic to clinical application and providing a foundation for subsequent applications.

Although logistic regression showed competitive ROC−AUC (0.780 vs 0.868) and identical Brier score (0.154) on the validation set, the stacking model offered two practical advantages. First, it achieved a more balanced trade−off between sensitivity (0.836 vs 0.745) and specificity (0.716 vs 0.758). In clinical practice, missing a high−risk BCRL patient (false negative) may lead to delayed intervention and worse outcomes, whereas a false alarm (low specificity) may only cause unnecessary monitoring. Therefore, the higher sensitivity of the stacking model is clinically advantageous when the cost of missing a case is high. Second, decision curve analysis (**Figure 5**) demonstrated a consistently higher net benefit for the stacking model across the clinically relevant threshold range (0.2–0.6), indicating that using the stacking model would lead to better clinical decisions than using the LR model or default ‘treat−all’/’treat−none’ strategies. Nevertheless, we acknowledge that the improvement over LR is modest, and the added complexity of stacking may not be justified in all settings, especially in resource−limited.

This study provides two main innovations. First, this is the first study in which controllable behavioral and lifestyle factors such as the daily time spent holding a mobile phone after surgery and patient’s awareness of BCRL were systematically incorporated into a BCRL risk prediction model, thereby improving the characteristic dimensions of risk assessment. Second, the StackBCRL interactive tool was developed and deployed online. This tool transformed the results of the stacking ensemble learning model into clinically applicable assessment results, thereby enhancing the clinical applicability of the research outcomes.

This study has several limitations. First, at the data and sample level, the stacking ensemble learning model constructed and validated in this study uses data from cross-sectional studies and single-center samples, which may introduce selection bias, thereby limiting the model’s application with different populations and medical institutions. Second, the validation set was derived from the same institution (single-center) and only separated by time (temporal validation). Therefore, this is not a true external validation. The reported performance metrics may be optimistic, and future studies should evaluate the model in independent, multi-center, and truly external cohorts. Third, the postoperative behavioral variables, including daily mobile phone use time after surgery, were self-reported and not validated against objective measures (e.g., accelerometer or smartphone logs). Recall bias and social desirability bias are possible. Although we adjusted for measured confounders (disease stage, rehabilitation compliance), residual confounding by unmeasured factors such as physical inactivity, arm posture, and socioeconomic status cannot be ruled out. The observed associations should not be interpreted as causal. Fourth, at the mechanism exploration and tool application level, although SHAP analysis can provide personalized risk explanations, the specific biological mechanisms and behavioral causal paths underlying the risk indicators identified by the model need to be clarified through prospective studies. Moreover, the clinical application value of StackBCRL, particularly its impact on clinical decision-making and patient prognosis, has not yet been verified and needs to be confirmed through prospective studies.

In conclusion, in this study, an interpretable stacking ensemble learning BCRL risk prediction model that integrates postoperative behavioral and lifestyles factors (the daily time spent using mobile phones after surgery and the patient’s awareness of BCRL) was constructed, and a StackBCRL visualization network tool was developed, enabling real-time prediction and visualization of individualized BCRL risks. Moreover, daily mobile phone use time after surgery and patient’s awareness of BCRL were identified as factors associated with BCRL risk in this cross-sectional analysis. These findings provide new research ideas for BCRL prevention and control, but causality should be tested in future prospective studies.

## Data Availability

The raw data supporting the conclusions of this article will be made available by the authors, without undue reservation.
